# The Hookworm Tissue Inhibitor of Metalloproteases (*Ac*-TMP-1) Modifies Dendritic Cell Function and Induces Generation of CD4 and CD8 Suppressor T Cells

**DOI:** 10.1371/journal.pntd.0000439

**Published:** 2009-05-19

**Authors:** Carmen Cuéllar, Wenhui Wu, Susana Mendez

**Affiliations:** 1 Departamento de Parasitología, Facultad de Farmacia, Universidad Complutense, Madrid, Spain; 2 J.A. Baker Institute for Animal Health, College of Veterinary Medicine, Cornell University, Ithaca, New York, United States of America; George Washington University, United States of America

## Abstract

Hookworm infection is a major cause of disease burden for humans. Recent studies have described hookworm-related immunosuppression in endemic populations and animal models. A Tissue Inhibitor of Metalloproteases (*Ac*-TMP-1) has been identified as one of the most abundant proteins released by the adult parasite. We investigated the effect of recombinant *Ac*-TMP-1 on dendritic cell (DC) and T cell function. Splenic T cells from C57BL/6 mice injected with *Ac*-TMP-1 showed reduced proliferation to restimulation with anti CD3 or bystander antigens such as OVA. Incubation of bone marrow-derived DCs with *Ac*-TMP-1 decreased MHC Class I and, especially, Class II expression but increased CD86 and IL-10 expression. Co-incubation of splenic T cells with DCs pulsed with *Ac*-TMP-1 induced their differentiation into CD4+ and, particularly, CD8+ CD25+Foxp3+ T cells that expressed IL-10. These cells were able to suppress proliferation of naïve and activated CD4+ T cells by TGF-Β-dependent (CD4+ suppressors) or independent (CD8+ suppressors) mechanisms. Priming of DCs with non-hookworm antigens, such as OVA, did not result in the generation of suppressor T cells. These data indicate that *Ac*-TMP-1 initiates the development of a regulatory response through modifications in DC function and generation of suppressor T cells. This is the first report to propose a role of suppressor CD8+ T cells in gastrointestinal helminthic infections.

## Author Summary

Chronic infections with helminths have been suggested to induce suppressor cells by a variety of mechanisms. The published immunological and epidemiological data on hookworm infection in humans and animal models suggest that these parasites are particularly successful in establishing chronicity and modulation. We have demonstrated that the recombinant form of a hookworm tissue inhibitor of metalloprotease (*Ac*-TMP-1) induces bone marrow-derived DCs to downregulate MHC Class I and II and release anti-inflammatory cytokines such as IL-10 and TGF-Β. More importantly, DCs pulsed with *Ac*-TMP-1 promote the development of regulatory CD4+ and, especially, CD8+ T cells from naïve T cells, which are able to suppress proliferation of naïve and activated splenic CD4+ T cells; this suppression is mediated by TGF-Β for CD4+ suppressor cells, but it is independent of the cytokine for CD8+ suppressor cells. These studies initiate the first steps to investigate the relevance and nature of suppressor T cells in hookworm infections and their mechanism of suppression.

## Introduction

The human hookworms *Necator americanus* and *Ancylostoma duodenale* are directly transmitted nematode parasites of the small intestine, and the main species that cause human hookworm infection, a leading cause of iron-deficiency anemia and malnutrition with a prevalence of 600 million cases in the tropical developing world [1]. Though mortality is rare, the global burden of hookworm disease is high, with an estimated 22 million Disability-Adjusted Life Years (DALYs) lost each year [1]. These numbers alone outrank diseases such as African trypanosomiasis, dengue, Chagas' disease, schistosomiasis, and leprosy [2].

For many common helminthic infections, including ascariasis, trichuriasis, and schistosomiasis, the intensity of infection peaks during childhood and adolescence [3]. In contrast, there appears to be considerable variation in the age profile of hookworm infection. Although the hookworm burden may be heavy in children, especially those in sub-Saharan Africa [4],[5], the most commonly recognized pattern is a steady rise in the intensity of infection during childhood, with either a peak or a plateau in adulthood. This lack of exposure or age-related immunity indicates that hookworms can either evade or suppress host immune responses. Studies performed by us and others have confirmed that hookworm infections decrease the ability of the immune system to respond to hookworm and bystander antigens, as evidenced by decreased lymphocyte responses in hookworm-infected humans [6],[7],[8], dogs [9] and hamsters [10],[11], as well as elevated serum IL-10 and immunosuppression in patients infected with *N. americanus* [12], or infected and exposed to adult parasite extracts [13]. Chemotherapy against the parasite restores the immune response in humans [14] and increases the immunogenicity of anti-hookworm vaccines in hamsters [10],[11].

Most of the pathology caused by the hookworm results from the adult stage of the parasite [15],[16]. While feeding, adult worms release into host tissues a battery of pharmacologically and immunologically active molecules [17]. Work by several groups has begun to unravel the biochemical events linked to the resultant blood loss that develops as a consequence of parasite attachment [18]. Among the secreted antigens, a hookworm-secreted Tissue Inhibitor of Metalloproteases (*Ac*-TMP-1) has been identified in *A. caninum* [19] and *A. ceylanicum* [20] as one of the most abundant proteins released by the adult parasite, at a rate of 40 ng/h [19].

In this report, we aimed to investigate the effect of the recombinant protein *Ac*-TMP-1 on dendritic cell function (DC) and generation of suppressor T cells. Splenic T cells from mice treated with *Ac*-TMP-1 exhibited decreased lymphoproliferative responses when restimulated ex vivo with Ac-TIMP or anti CD3. To understand the mechanism behind this suppression of proliferation, we incubated bone marrow-derived dendritic cells (DCs) from C57BL/6 mice (B/6) with *Ac*-TMP-1, and discovered that DCs exposed to the hookworm antigen decreased expression of MHC Class I and II and increased expression of CD86 and IL-10, as well as production of TGF-Β. Moreover, co-incubation of naïve splenic T cells with DC pulsed with *Ac*-TMP-1 induced their differentiation of T cells into IL-10 producing CD4+ and CD8+ CD25+Foxp3+ regulatory T cells that suppressed proliferation of both naïve and activated CD4+ T cells. Interestingly, neutralization of the cytokine TGF-Β reduced the suppressive ability of *Ac*-TMP-derived CD4+ T cell suppressors, but did not affect the ability of the CD8+ cells to suppress proliferation. Because CD4, but particularly CD8+ T cells are abundant in the gut (the site of hookworm infection) we propose a novel mechanism of immunosuppression by a parasitic helminth.

## Materials and Methods

### Recombinant *Ac*-TMP-1

Recombinant *Ac*-TMP-1 was kindly provided by Dr. Bin Zhan and Dr. Peter Hotez at The George Washington University. To generate the recombinant protein, a cDNA encoding a putative tissue inhibitor of metalloproteinase was cloned from an *Ancylostoma caninum* adult hookworm cDNA library by immunoscreening with anti-hookworm secretory products antiserum. The protocol of the cloning and protein expression is described in detail elsewhere [19].

### Mice

C57BL/6 (B/6) mice were purchased from Taconic (Germantown, NY). All mice were maintained in the Baker Institute Animal Care Facility under pathogen-free conditions. All animal studies were approved by the Institutional Animal Care and Use Committee at Cornell University.

### *In vivo* treatment with *Ac*-TMP-1 of C57BL/6 mice

We simulated continuous exposure by injecting 50 µg *Ac*-TMP-1 or ovoalbumin (OVA) to C57BL/6 mice intraperitoneally, every 2 days, for a total of 8 days. Two days after the last injection, spleens were collected, T cells purified with enrichment columns (R& D Systems, Minneapolis, MN), labeled with CFSE as described [10] and restimulated with 50 µg *Ac*-TMP-1 or 5 µg/ml anti CD3. Cells were harvested 5 days later. Proliferation was assessed by loss of CFSE staining.

### *In vitro* bone marrow-derived DC stimulation assays

Bone marrow-derived DCs were cultured in the presence of 20 ng/ml GM-CSF and collected 6–8 days after culture. DCs were then plated in 6-well plates (10^6^/well) before *Ac*-TMP-1 was added to the wells. At different time points, brefeldin A (10 µg/ml) was added for 6 h and DCs were then collected and fixed in 4% paraformaldehyde. Prior to staining, cells were incubated with an anti-Fcγ III/II receptor and 10% normal mouse serum (NMS) in PBS containing 0.1% BSA, 0.01% NaN_3_. Cells were permeabilized and stained for the surface markers CD11c (clone 223H7), CD80 (clone 16-10A1), CD86 (clone GL1), MHC Class I (clone 28-14-8) and MHC Class II (clone M5/114.15.2) and for the cytokines IL-12p40/p70 (clone C17.8) and IL-10 (clone JES5-16E3). Incubations were carried out for 30 min on ice. Unless specified, all antibodies were purchased from BD Biosciences or eBioscience. The data were collected using a FACScalibur flow cytometer and analyzed in CELLQuest software (Becton Dickinson, San Jose, CA). For each sample, at least 30,000 cells were analyzed.

### TGF-Β detection by ELISA

ELISAs for the detection of murine TGF-Β were carried out using antibodies from R&D systems (Minneapolis, MN) following the manufacturer's instructions.

### Phenotypic and functional characterization of T cells co-cultured with *Ac*-TMP-pulsed bone marrow-derived DCs

Splenocytes were purified from spleens of naive B/6 mice by mechanical disruption. Prior to co-culture, red blood cells were lysed for 10 minutes with cold ACK lysing buffer. T cells were enriched using columns as above, and added to cultures containing bone marrow-derived DCs (5 T cells: 1 DC ratio) that have been left unstimulated or had been treated with *Ac*-TMP-1 (50 µg for 16 h). Forty-eight hours after initiation of the co-culture, brefeldin A was added for 6 h; cells were then collected and fixed in 4% paraformaldehyde. Prior to staining, cells were incubated with an anti-Fcγ III/II receptor and 10% normal mouse serum (NMS) in PBS containing 0.1% BSA, 0.01% NaN_3_. Cells were permeabilized with saponin and stained for the surface markers CD4, (clone RM4-5), CD8 (clone 53-6.7), CD25 (clone PC61.5), the transcription factor Foxp3 (FJK-16s), IFN-γ (clone XMG1.2) and IL-10 (clone JES5-16E3). Incubations were carried out for 30 min on ice. All antibodies were purchased from BD Biosciences or eBioscience. The data were collected and analyzed by flow cytometry as described above.

### Suppression of proliferation assay

Suppressor cells (CD4+ and CD8+) used for this assay were generated by incubation with bone marrow-derived DCs pulsed with 50 µg *Ac*-TMP-1 as described above. Following priming and seeding of DCs, they became adherent and cannot be easily collected from the experimental well. T cell purity was determined following collection by cytospins, Diff quick staining and quantitation under the microscope. The percentage of T cells collected from the wells containing DCs was >98%. Target cells (CD4+ T cells) were isolated from the spleens of B/6 using magnetic beads (Miltenyi, Auburn, CA) [10]. Naïve T cells were stained with CFSE and used immediately. Activated CD4 T cells were generated by culture with anti-CD3 (5 µg/ml) for 3 days, when they were also labeled with CFSE. The CD4+ or CD8+ suppressor cells were treated with 0.8 µg/ml mitomycin C (Sigma-Aldrich, St. Louis, MO) to prevent their proliferation and added to the CFSE labeled target cells CD4+ T cells, at a 1∶1 ratio. To trigger proliferation of target cells, anti-CD3 (5 µg/ml) and IL-2 (10 U/ml) were added. Cells were harvested, and proliferation was measured at day 5. Neutralization of IL-10 was performed employing anti-mouse IL-10 neutralizing antibody (R&D systems) at a concentration of 10 ng/ml. Neutralization of TGF-Β was carried out by adding anti-mouse TGF-Β neutralizing antibody (R&D systems) at a concentration of 25 µg/ml.

### Statistics

Data are presented as mean±SD or SEM. Differences were analyzed for significance Student's unpaired, two-tailed *t*-test or ANOVA using Graph Pad Prism Software (San Diego, CA). A P value less than 0.05 was used as the threshold for significance. Specific P values are indicated in each figure.

## Results

### *In vivo* treatment of mice with *Ac*-TMP-1 decreased the ability of their splenic T cells to proliferate *ex vivo*

**Figure 1:**
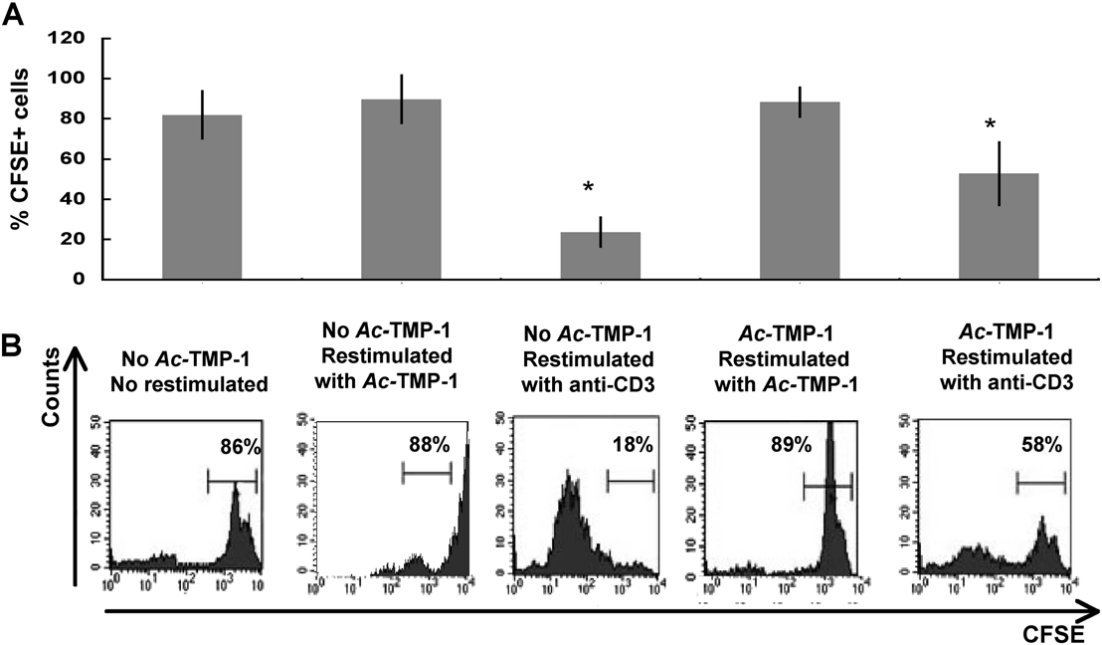
Decreased lymphoproliferative responses in mice treated with *Ac*-TMP-1. A. Proliferation profile of CSFE-labeled splenic T cells from B/6 mice treated with PBS, *Ac*-TMP-1 or OVA and restimulated *ex vivo* with 50 µg/ml *Ac*-TMP-1, 50 µg/ml OVA or 5 µg/ml anti CD3. FACS histograms are gated on CD4+ cells and show the intensity of CFSE staining 5 days after restimulation. Numbers shown indicate percentage of cells in the region depicted in the histogram. Data show a representative experiment of 3 independent determinations. B. Percentage of CFSE+CD4+ splenic T cells from mice cells from B/6 mice treated with PBS, *Ac*-TMP-1 or OVA and restimulated *ex vivo* with *Ac*-TMP-1, OVA or with anti CD3. Data are expressed as average±SEM (n = 3). *: Statistically significant, P = 0.002.

We wanted to explore the effect of *Ac*-TMP-1 exposure in a small animal model. The mouse is not a permissive model of hookworm infection, because the parasite cannot establish itself in the gut. Thus, we simulated continuous exposure by injecting 50 µg *Ac*-TMP-1 to B/6 mice intraperitoneally, every 2 days, for a total of 8 days. This regimen was substantiated by our knowledge of the parasite and the pharmacokinetics of the intraperitoneal route. An adult hookworm secretes 40 ng *Ac*-TMP-1/h [21]. Because adult infections typically range from 10–100 worms [22], the level of *Ac*-TMP-1 in the interstitial fluid/serum at any given time should be 0.4–4 µg. Drugs given intraperitoneally have a half life in serum of 30–40 h, and a recovery of 1–10% of the original concentration. By injecting every 2 days, *Ac*-TMP-1 would always be at the maximum concentration in serum (0.5–5 µg), simulating infection conditions. As controls, we injected either PBS or the non-hookworm protein ovoalbumin (OVA) using the same regimen. Two days after the last injection, spleens were collected, T cells purified, labeled with CFSE and restimulated *ex vivo* with *Ac*-TMP-1, OVA or anti CD3 for 5 days. Proliferation (or lack thereof) was assessed by determining the percentage of CFSE-positive cells. Figure 1A shows that unstimulated cells did not proliferate in culture (86–90% did not lose CFSE staining). Interestingly, mice did not proliferate in response to *Ac*-TMP-1 restimulation, including the mice that were primed with the antigen *in vivo*. As expected, T cells from control mice that had been injected with PBS or OVA proliferated in response to anti CD3 (only 18–25% retained CFSE staining, P = 0.002). *Ex vivo* proliferation to anti CD3 was decreased in mice treated with *Ac*-TMP-1 when compared to PBS-injected control animals (58 *vs.* 18% cells positive for CFSE, P = 0.002). Most strikingly, proliferation to OVA was decreased in OVA-primed mice if cells were restimulated *ex vivo* in the presence of *Ac*-TMP-1. This experiment was repeated 3 times and the average±SEM is shown in Figure 1B. These data indicate that *in vivo* treatment of mice with *Ac*-TMP-1 decreases the ability of their splenic T cells to initiate lymphoproliferative responses to the hookworm protein, or bystander antigens.

### Incubation of bone marrow-derived DCs with *Ac*-TMP-1 induced changes in the expression of activation markers and cytokines

**Figure 2:**
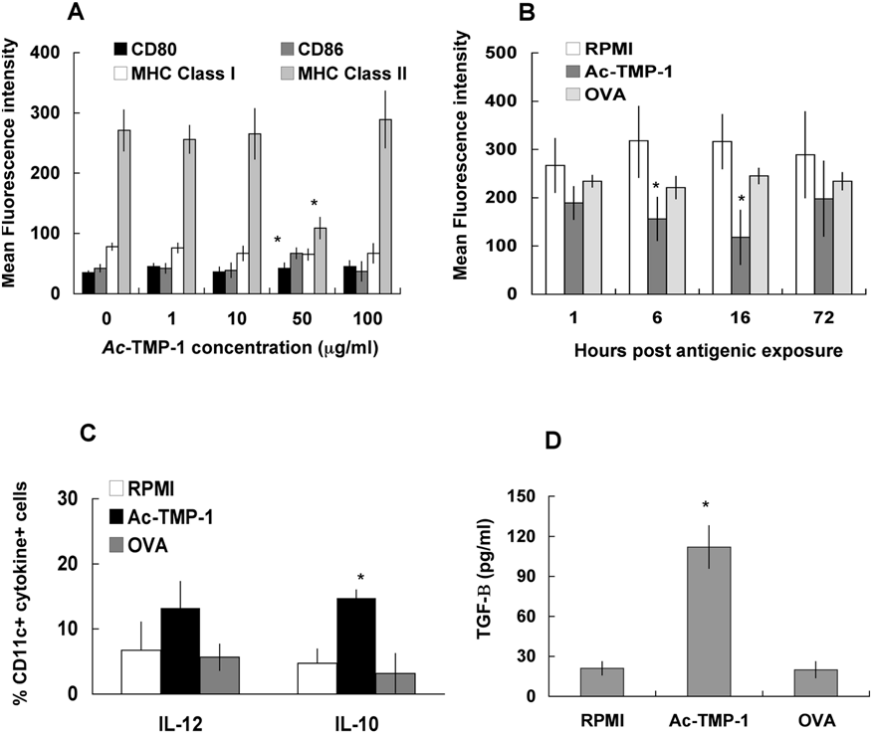
*Ac*-TMP-1 treatment affects their phenotype and cytokine expression bone marrow-derived dendritic cells. A. Flow cytometry profiles of CD80, CD86 and MHC Class I and II expression levels in CD11c+ bone marrow-derived DCs incubated for 16 hours with RPMI or 1–100 µg recombinant *Ac*-TMP-1. Data are expressed as average±SEM of 3 different experiments. *: Statistically significant, P = 0.02. B. Flow cytometry profiles of MHC Class II expression levels in DCs incubated at different time points with RPMI, 50 µg recombinant *Ac*-TMP-1 or 50 µg OVA. Data are expressed as average±SEM of 3 different experiments. *: Statistically significant, P = 0.008. C. Intracellular cytokine staining for IL-12p40/p70 and IL-10 in CD11c+ bone marrow-derived DCs unstimulated or stimulated with *Ac*-TMP-1 or OVA. Data are expressed as average±SEM of 4 different experiments. *: Statistically significant, P = 0.001. D. Secretion of TGF-Β in supernatants of DCs primed with the antigens as in B. Data are expressed as average±SEM of 3 different determinations. *: Statistically significant, P = 0.0001.

In order to elucidate the mechanism(s) of immunosuppression by *Ac*-TMP-1, we turned into *in vitro* models of DC-T cell interactions. To optimize *in vitro* conditions, bone marrow-derived DCs from B/6 mice were obtained and cultured in RPMI or in the presence of increasing doses (1–100 µg) *Ac*-TMP-1 for 16 hours, when the cells were collected and fixed. The Mean Intensity of Fluorescence (MFI) in CD11c+ DCs expressing the co-stimulatory molecules CD80 and CD86, as well as MHC Class I and II was analyzed by flow cytometry (Figure 2A). In the presence of *Ac*-TMP-1, MFIs for CD80 and CD86 were slightly increased, and the difference was statistically significant (P = 0.02) for the latter activation marker if exposed to 50 µg *Ac*-TMP-1. MFI values for MHC Class I decreased (78 in unstimulated cells *vs.* 51 in *Ac*-TMP-1-treated cells, although the difference was not statistically significant). In contrast, MHC Class II MFI was significantly downregulated (P = 0.008) by incubation with 50 µg *Ac*-TMP-1. Because this concentration was the dose at which the highest effect was observed, we generated a time-course curve in which the level of MHC Class II expression was detected at 1, 6, 16 and 72 h. In this experiment, we included a non-hookworm protein as a control, and incubated DCs with 50 µg/ml OVA. Figure 2B shows that the expression of the surface marker was already decreased since after 1 h incubation with the hookworm antigen and onwards. The downregulation of expression was statistically significant at 6 and 16 h post antigenic exposure when compared to RPMI-treated cells. No changes were observed in OVA-primed DCs. These results indicated that 50 µg *Ac*-TMP-1 was able to induce a biological effect on DCs in a period between 6–16 h.

Intracellular staining for the cytokines IL-12p40/p70 and IL-10 was also determined in DCs stimulated with *Ac*-TMP-1 or OVA for 16 h (Figure 2C). IL-12 expression was slightly increased following antigenic stimulation, although the difference with the RPMI-treated control was not statistically significant. In contrast, IL-10 expression was significantly increased (P = 0.001). Cytokine data assayed by ELISA confirmed the results (not shown). Priming with OVA did not induce a cytokine response. Finally, the levels of the anti-inflammatory cytokine TGF-Β were determined in the DC supernatants by ELISA. The secretion of TGF-Β was increased in DC cultures incubated with *Ac*-TMP-1, and unaffected by OVA. Together, these results indicate that *in vitro* treatment of bone marrow DCs with *Ac*-TMP-1 decreased their ability to present antigen and increased their ability to produce anti-inflammatory cytokines such as IL-10 and TGF-Β.

### Co-incubation of splenic T cells with bone marrow-derived DCs pulsed with *Ac*-TMP-1 induced the generation of CD25+Foxp3+ IL-10+ suppressor T cells

**Figure 3:**
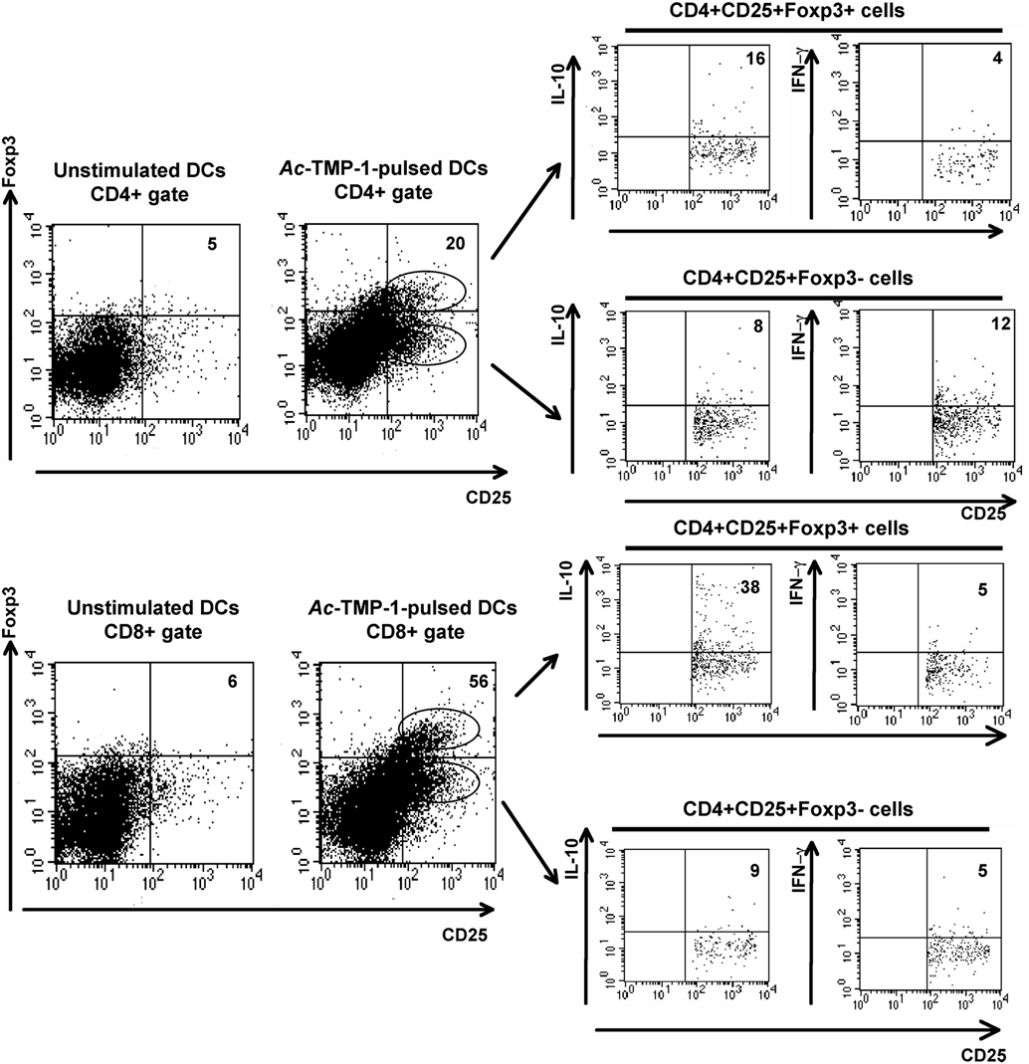
*Ac*-TMP-1 induces generation of regulatory T cells. Splenic T cells were co-cultured with bone marrow-derived DCs treated with *Ac*-TMP-1 (50 µg) for 16 h at a T cell: DC ratio of 5∶1. Cells were gated by CD4 or CD8. The percentage of CD25+ expression Foxp3 was first analyzed in the CD4 (upper left) and CD8 (lower left) populations. Then, the expression of IFN-γ or IL-10 in the CD25+Foxp3− or CD25+Foxp3+ populations was determined (right) and indicated at the upper right corner of each histogram. Data shown are representative from 3 independent experiments.

For this experiment, splenic T cells were obtained from B/6 mice and co-cultured for 48 h with either unstimulated or *Ac*-TMP-1-treated bone marrow-derived DCs (for 6 h with 50 µg *Ac*-TMP-1). DCs alone produced negligible amounts of cytokines (<2%, not shown). First, we determined the percentage of CD4+ and CD8+ T cells expressing the activation marker CD25 and the transcription factor Foxp3 (expressed in regulatory T cells). *Ac*-TMP-1 priming increased Foxp3 expression in CD4+ T cells (from 5% to 20%) and especially, in CD8+ T cells (from 6% to 56%) (Figure 3), demonstrating that the hookworm antigen induced naïve T cells to become regulatory T cells. This finding was further confirmed by the study of cytokines. We determined the expression of IFN-γ and IL-10 in both the CD25+Foxp3− (activated, non-regulatory T cells) and CD25+Foxp3+ lymphocyte populations (true regulatory T cells). Cytokine expression in unstimulated controls was <5% (not shown). *Ac*-TMP-1 treatment induced CD4+ CD25+Foxp3− cells to express IFN-γ (to 12%); in contrast, IFN-γ expression in CD4+CD25+ Foxp3+ cells was only 4%. The frequency of both CD8+CD25+ Foxp3+IFN-γ+ and CD8+ CD25+Foxp3−IFN-γ+ cells was low (5%). Both CD4+ and CD8+ CD25+ T cells co-cultured with *Ac*-TMP-1 treated DCs expressed IL-10 (8% and 9% respectively). Interestingly, *Ac*-TMP-1-treated bone marrow-derived DCs induced the highest increase in IL-10 expression in CD4+ CD25+Foxp3+ cells (to 16%) and, most strikingly, in the CD8+CD25+Foxp3+ population (38%). These findings show that *Ac*-TMP-1-treated bone marrow-derived DCs selectively biased the differentiation of naive T cells, in particular CD8+ T cells, toward a regulatory phenotype *via* increased expression of the transcription factor Foxp3 and the cytokine IL-10.

### *Ac*-TMP-1-primed CD4+ and CD8+ cells suppress proliferation of naïve and activated CD4+ T cells *via* TGF-Β dependent and independent mechanisms

For these experiments, target CD4+ T cells were purified from the spleens of naïve B/6 mice. Activated CD4+ T cells were generated by restimulation *in vitro* with anti-CD3 for 3 days. Both were labeled with CFSE, and plated. The suppressor T cells were generated by incubation of splenic naïve T cells with bone marrow-derived DCs pulsed with *Ac*-TMP-1, OVA or RPMI (unstimulated controls). Suppressor CD4+ and CD8+ T cells were then added to the target naïve or activated CFSE-stained CD4+ T cells. Co-cultures were then incubated with a mixture of anti CD3/IL-2 to enhance proliferation of target T cells in the presence or absence of neutralizing antibodies for IL-10 and TGF-Β. Negative proliferation was quantitated as % CFSE+ cells 5 days after initiation of co-culture.

**Figure 4:**
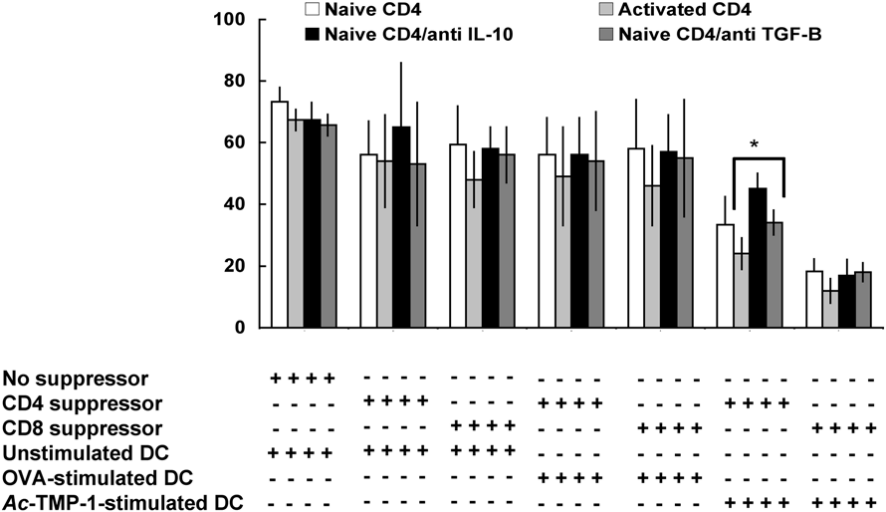
*Ac*-TMP-1-induced suppressor cells decrease proliferation of CD4+ T cells. Percentage of CFSE+ cells in naïve and activated CD4+ T cell cultures following co-culture with suppressor T cells. Suppressor CD4+ or CD8+ T cells were generated through incubation with unstimulated or either OVA or *Ac*-TMP-1-primed bone marrow-derived DCs. Suppressor T cells were co-incubated with CFSE-labeled naïve or anti CD3 activated CD4+ T cells and restimulated with anti CD3 and IL-2. In some wells, neutralizing antibodies against either IL-10 or TGF-Β were added. The percentage of CFSE+ cells was determined in the target population by flow cytometry. Figure shows are the mean±SD from 3 independent determinations. *: Statistically significant, P = 0.05.

In the absence of suppressor T cells, >70% cells proliferated in response to antiCD3/IL-2 treatment (Figure 4); the proliferation was unaffected by cytokine neutralization. Moreover, >70% of the naïve CD4+ T cells proliferated following co-culture with CD4+ and CD8+ T cells incubated with unstimulated DCs. Again, neutralization of IL-10 or TGF-Β did not produce any effect. Similarly, OVA-primed T cells were unable so suppress proliferation of splenic T cells. *Ac*-TMP-1 primed-CD4+ T cells were able to suppress the proliferation of naïve CD4+ T cells (to 35%) and activated CD4+ T cells (to 25%), although the difference was not statistically significant when compared to unstimulated cells alone. Treatment of cultures with anti-IL-10 antibodies resulted in a decrease in the ability of *Ac*-TMP-1 primed CD4+ T cells to suppress proliferation (from 35 to 48% CFSE+ cells, although the difference was not statistically significant. However, the decrease in suppressive ability in cultures where TGF-Β was neutralized was statistically significant (P = 0.05). Finally, CD8+ T cells primed with *Ac*-TMP-1 were more effective in suppressing T cell responses by significantly decreasing the ability of both naïve and activated CD4+ T cells to divide (to 20% and 12%, respectively). The suppressive ability of these cells was not abolished by neutralization of either IL-10 or TGF-Β. These results demonstrate that DC priming with *Ac*-TMP-1, but not other proteins, induced the generation of CD4+ and CD8+ suppressor T cells. In our system, CD8+ suppressor T cells were more efficient in reducing proliferation of both naïve and activated target CD4 T cells, and their ability to suppress was unaffected by neutralization of either IL-10 or TGF-Β, as opposed as the CD4+ suppressors, that required both cytokines, in particular TGF-Β.

## Discussion

Chronic infections with helminths have been suggested to induce suppressor cells by a variety of mechanisms. The published immunological and epidemiological data on hookworm infection in humans and animal models suggest that these parasites are particularly successful in establish chronicity and modulation [1],[6],[7],[10],[11],[17],[23],[24],[25],[26],[27],[28]. Numerous helminth-derived proteins are though to contribute to the immunosuppression associated with these parasites [29],[30]. Tissue inhibitors of metalloproteases themselves have proven to have immunomodulatory properties [31],[32],[33],[34]. In this report, we have investigated the effect of the hookworm tissue inhibitor of metalloproteases *Ac*-TMP-1, one of the most abundant proteins released by the parasite following establishment, on DC function and T cell differentiation. We have demonstrated that recombinant *Ac*-TMP-1 is able to induce bone marrow-derived DCs to downregulate MHC molecules and release anti-inflammatory cytokines. More importantly, DCs pulsed with *Ac*-TMP-1 promoted the development of regulatory T cells. Remarkably, CD8+ suppressor T cells were more abundant, more potent, and used different suppressive mechanisms than CD4+ T cells.

It is considered that the immature developmental stages of DC differentiation produce tolerogenic DCs which in turn induce T cell anergy or regulatory T cells [35]. The controlled environment of the *in vitro* experiments performed by us revealed that bone marrow-derived DCs decreased their ability to present antigen (by downregulating MHC Class I and, especially class II expression) and increased their ability to produce the anti-inflammatory cytokines IL-10 and TGF-Β. This phenotype is consistent with the development of tolerogenic DCs [36]. Thus, the initiation of suppressive responses in hookworm infectious may be initiated by an increased frequency in the tolerogenic DC population in the sites where the antigen is released. How CD8+ suppressor T cells generate after the first interaction with DCs is still unknown. Whereas downregulation of MHC Class I has been implicated in the generation of suppressor T cells by some, others have proposed that their generation do not require MHC mechanisms, or that it may be caused by the recognition of other ligands, such as CD40L [37],[38],[39],[40]. While our studies demonstrate the generation of a suppressive population of T cells, the exact mechanism whereby these cells arise deserves further investigation.

Studies in human populations and animal models have suggested that adult hookworms are immunosuppressive. In fact, our experiments in animal models have revealed that peripheral blood or splenic cells from dogs and hamsters infected with the parasite do not proliferate in response to adult hookworm extracts [9],[11]. Recently, the analysis of human responses to adult hookworm extracts demonstrate that restimulation of peripheral blood cells with adult proteins causes an increase in IL-10 production [13]. The *in vivo* studies presented here support this hypothesis and demonstrate that priming mice with the abundant adult extract protein *Ac*-TMP-1 results in a decrease their lymphoproliferative responses to TCR stimulation. More importantly, they also revealed that exposure to *Ac*-TMP-1 also diminished specific proliferation to bystander antigens, such as OVA, suggesting that the hookworm antigen is able to cause potent, generalized immunosuppression. This is further demonstrated by the fact that mice exposed to *Ac*-TMP-1 are unable to initiate lymphoproliferative responses to the antigen. Interestingly, dogs vaccinated against *Ac*-TMP-1 did not develop proliferative responses against the hookworm antigen (unpublished).

Our *in vitro* experiments attempt to begin to unravel the underlying mechanism of immunosuppression and revealed that priming DCs with *Ac*-TMP-1 induced the *de novo* generation of CD4+, and more importantly, CD8+ Foxp3+ IL-10+ cells. Both cell populations are able to display suppressive functions when co-cultured with both CD4+ naïve and activated T cells. Interestingly, the mechanism of suppression by CD4+ suppressor T cells seems to be mediated by the release of anti-inflammatory cytokines, whereas CD8+ suppressor T cells do not require the presence of IL-10 or TGF-Β to suppress T cell proliferation, suggesting perhaps direct cell contact mechanisms. The existence of suppressor CD8 populations has been documented in different models [41],[42], although their role and mechanism of suppression remains poorly characterized. Whereas some authors postulate that their suppressive function is dependent on IL-10 production, some others demonstrate that their function is cytokine independent (*i.e.* [38],[39]). Although our data support the latter hypothesis, further experiments need to be performed to determine the mechanism of immunosuppression in *Ac*-TMP-1-primed suppressor T cells, and the relevance of such mechanism in infection models. Because the role of CD8+ suppressor T cells has been postulated as a very important homeostatic mechanism in the gut mucosa [43], the infection site for hookworms, the demonstration of the role of CD8+ suppressor T cells in the regulation of gut immunity will be of importance not only to bring forward the role of these cells in the mucosal environment, but also to enhance the relevance of this effector population in the context of gastrointestinal parasitic infections. CD8 suppressor T cells have been generated *in vitro* in response to other extracellular nematodes such as *Echinococcus multilocularis* protoscoleces [44], but this is the first report in which they have been implicated in the immune response against gastrointestinal nematodes.

In summary, our data demonstrates that *Ac*-TMP-1 modulates the immune response of the host by more than one mechanism(s). This hookworm molecule appears to induce de-activation of the DC and enhance IL-10 production, and to elicit the development of T cells with regulatory functions. These findings open the door to future studies to determine the nature of the interaction of the hookworm antigen with antigen presenting cells, as well as to investigate the relevance of suppressor T cells in helminthic infections, and their mechanism of suppression.
